# Genome-wide screening of meta-QTL and candidate genes controlling yield and yield-related traits in barley (*Hordeum vulgare* L.)

**DOI:** 10.1371/journal.pone.0303751

**Published:** 2024-05-20

**Authors:** Binbin Du, Jia Wu, Qingming Wang, Chaoyue Sun, Genlou Sun, Jie Zhou, Lei Zhang, Qingsong Xiong, Xifeng Ren, Baowei Lu

**Affiliations:** 1 College of Biotechnology and Pharmaceutical Engineering, West Anhui University, Lu’an, China; 2 Lu’an Academy of Agricultural Science, Lu’an, China; 3 Biology Department, Saint Mary’s University, Halifax, Canada; 4 Zoomlion Intelligent Agriculture Co. Ltd., Wuhu, China; 5 Hubei Hongshan Laboratory, College of Plant Science and Technology, Huazhong Agricultural University, Wuhan, China; Texas A&M University and Texas A&M Agrilife Research, UNITED STATES

## Abstract

Increasing yield is an important goal of barley breeding. In this study, 54 papers published from 2001–2022 on QTL mapping for yield and yield-related traits in barley were collected, which contained 1080 QTLs mapped to the barley high-density consensus map for QTL meta-analysis. These initial QTLs were integrated into 85 meta-QTLs (MQTL) with a mean confidence interval (CI) of 2.76 cM, which was 7.86-fold narrower than the CI of the initial QTL. Among these 85 MQTLs, 68 MQTLs were validated in GWAS studies, and 25 breeder’s MQTLs were screened from them. Seventeen barley orthologs of yield-related genes in rice and maize were identified within the hcMQTL region based on comparative genomics strategy and were presumed to be reliable candidates for controlling yield-related traits. The results of this study provide useful information for molecular marker-assisted breeding and candidate gene mining of yield-related traits in barley.

## Introduction

Barley is one of the fourth largest cereal crops first domesticated globally [1]. Barley remains an important cereal in many regions, such as North Africa, Asia, and parts of South America. With increasing global climate change and rising food demand, improving barley yield is critical. Barley yield is a complex quantitative trait controlled by numerous minor genes and is susceptible to environmental influences [2]. Yield is mainly composed of the number of spikes, number of grains per spike, and thousand grain weight, while plant height, number of tillers, biomass, and harvest index have significant effects on barley yield [3–6]. In addition, the growing periods such as the heading date, flowering date, grain filling period, and maturity stage are also important factors affecting yield in barley [7, 8].

Traditional quantitative trait loci (QTL) mapping for complex yield-related traits mainly used biparental segregating populations, then used molecular marker-assisted selection to accelerate the process of breeding new varieties with superior quality and high yield [9, 10]. However, QTL results lacked consistency and wide applicability for use in barley breeding processes, limited by differences in genotypes and environmental conditions [11]. Meta-analysis was first developed by Goffinet and Gerber [12] to integrate and map QTL from different trials into a consensus map, narrowing the confidence interval of QTL to obtain stable and reliable meta-QTL (MQTL). MQTL excludes the interference of specific environments, genotypes, and molecular marker types, and has been used in crop breeding and yield-related trait improvement [13].

Recently, QTL meta-analysis has been widely used for QTL integration of complex quantitative traits in different crops, such as ionome-related traits in *Arabidopsis thaliana* [14], grain water content, grain dehydration rate and yield-related traits in maize [15, 16], grain weight and yield-related traits in rice [17, 18], grain zinc and iron contents, flag leaf morphology, quality and yield-related traits in wheat [19–21]. In addition, QTL meta-analysis has also been reported for several traits in barley, such as three studies on abiotic stress tolerance traits [11, 22, 23], and one on yield and yield-related traits [24], in which a total of 31 MQTLs were identified, but limited by the number of QTL mapping populations and the initial QTL, the results have certain limitation and need further refinement.

Besides conventional QTL linkage analysis, benefiting from the development of high-throughput genotyping technology, genome-wide association studies (GWAS) based on natural populations have been used to identify MTA for complex quantitative traits in rice, wheat, brassica napus and other crops [25–28]. With the mutual validation of the results of linkage analysis and GWAS, major QTL or candidate genes affecting deep root and peduncle vascular bundle-related traits were identified in rice [29, 30], and key genomic regions controlling spike-related traits and root length traits were identified in wheat [31, 32]. These studies suggest that GWAS can facilitate the validation of the accuracy of QTL meta-analysis for the mining of QTL or candidate genes associated with important quantitative traits.

In this study, we exhaustively collected QTL for 11 yield-related traits such as barley grain yield, grain morphology, grain number, spike-related traits, grain weight, growth period traits, plant height, tiller number, etc. published in the last two decades for QTL meta-analysis, to identify more MQTLs affecting yield. The accuracy of MQTL was further validated using GWAS and available transcriptome results to identify genomic regions and candidate genes associated with yield in barley. This study will enhance our understanding of the genetic mechanism for yield and yield-related traits in barley, and provide a basis for molecular marker-assisted selection and aggregation of important yield QTL or genes in barley.

## Results

### QTL studies for yield and yield-related traits in barley

**Fig 1:**
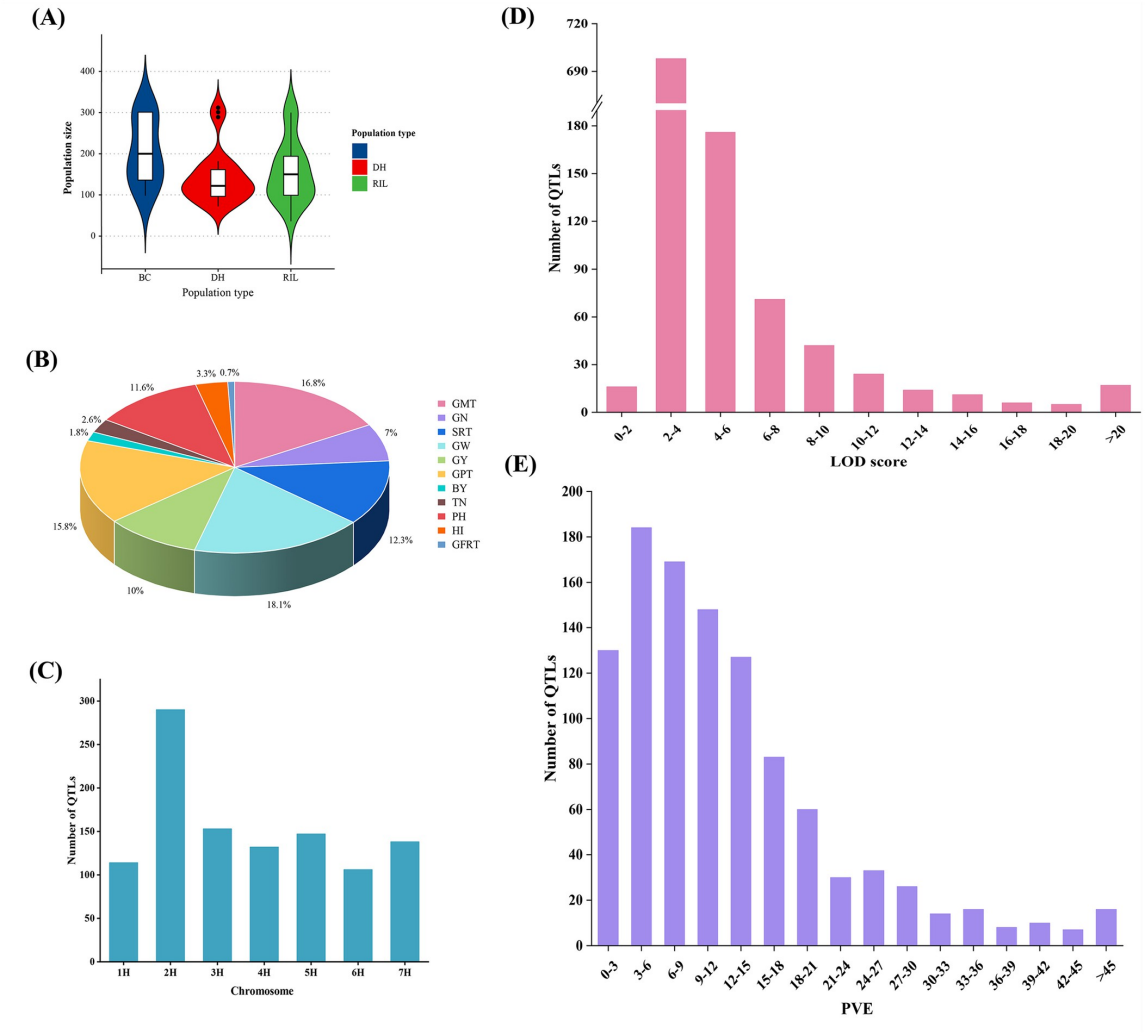
QTL information for barley yield and yield-related traits in previous QTL studies. (A) Population size of different population types. (B) Percentage of QTL for different yield and yield-related traits. (C) QTL distribution on 7 chromosomes. (D) Frequency distribution of QTL with different LOD scores. (E) Frequency distribution of QTL for different PVEs.

Fifty-four previous QTL studies on yield-related traits in barley from 2001–2022 were analyzed, and details were listed in the S1 Table. A total of 80 mapping populations were involved in the 54 studies, including 10 backcross (BC) populations, 27 double haploid (DH) populations, and 43 recombinant inbred line (RIL) populations with population sizes ranging from 28–301, 72–312, and 35–300, respectively (Fig 1A; S1 Table).

A total of 1080 QTLs were identified for barley yield and related traits in 80 mapping populations from the 54 studies (S2 Table). The methods of measuring the same or similar traits differed in certain studies, such as the number of spikes expressed as spikes per plant, spikes per square meter, and spikes per line. Through comparative screening, these traits were classified into 11 main types, including grain morphological traits (GMT), grain number (GN), spike-related traits (SRT), grain weight (GW), grain yield (GY), grain filling-related traits (GFRT), growth period traits (GPT), biomass yield (BY), plant height (PH), tiller number (TN) and harvest index (HI). GN, SRT, GW, and GY, as the major yield traits, accounted for 47.4% of the number of initial QTL; GMT, GPT, and PH occupied 44.2% of QTL, which were important factors affecting barley yield; BY, TN, HI, and GFRT accounted for 8.4% of initial QTL (Fig 1B). These initial QTLs were unevenly distributed on seven chromosomes of barley, with the highest number of QTL on chromosome 2H, accounting for 26.85% of the total (290/1080), and the remaining chromosomes accounting for 9.81% to 14.17% of the total (Fig 1C). The LOD scores of the initial QTL varied between 1.02 and 70.47, 65.19% of the QTL had LOD scores in the range of 2 to 4 (Fig 1D). The PVE of individual QTL ranged from 0.26% to 91.32%, and the 183, 169, and 148 QTLs had PVE ranging from 3–6%, 6–9% and 9–12%respectively, accounting for 46.27% of the total (Fig 1E).

### Construction of a high-density consensus map in barley

**Fig 2:**
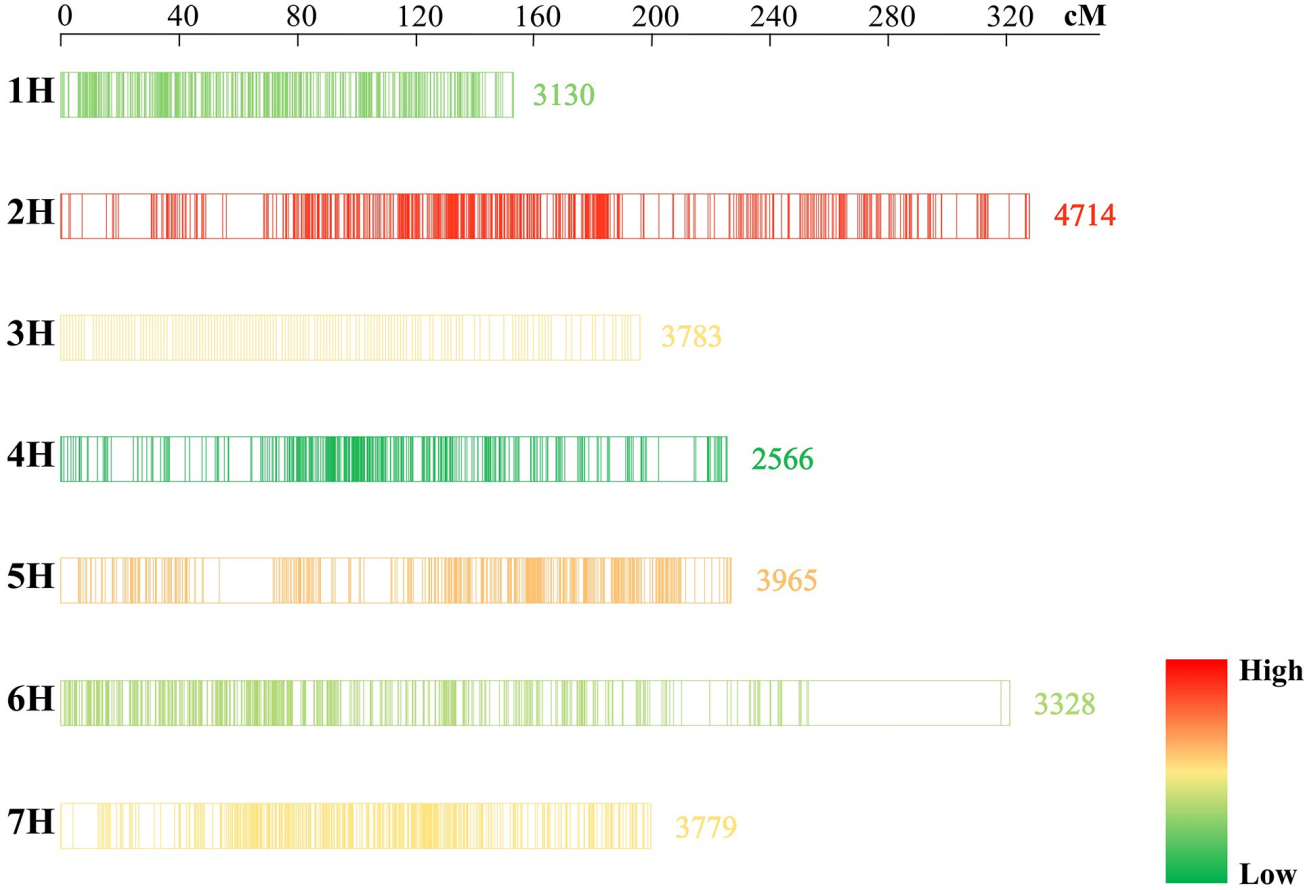
Distribution of markers on the barley consensus map in this study. The number of markers decreases sequentially from red to green.

A consensus map containing DArT, SSR, RFLP, SNP markers, and a few genes was constructed using the R package LPmerge [33]. The map contained 25265 markers with a total length of 1650.43 cM, and the genetic length of individual chromosomes varied from 153.28 cM on 1H to 328.04 cM on 2H, with an average chromosome length of 235.78 cM (S3 Table). The number of markers on different chromosomes varied from 2566 on 4H to 4714 on 2H, and the marker density on each chromosome ranged from 10.36 (6H) to 20.42 (1H) per cm, and the average genetic distance between two markers on the whole map was 0.07 cM (Fig 2; S4 Table).

### QTL meta-analysis for yield and yield-related traits

**Fig 3:**
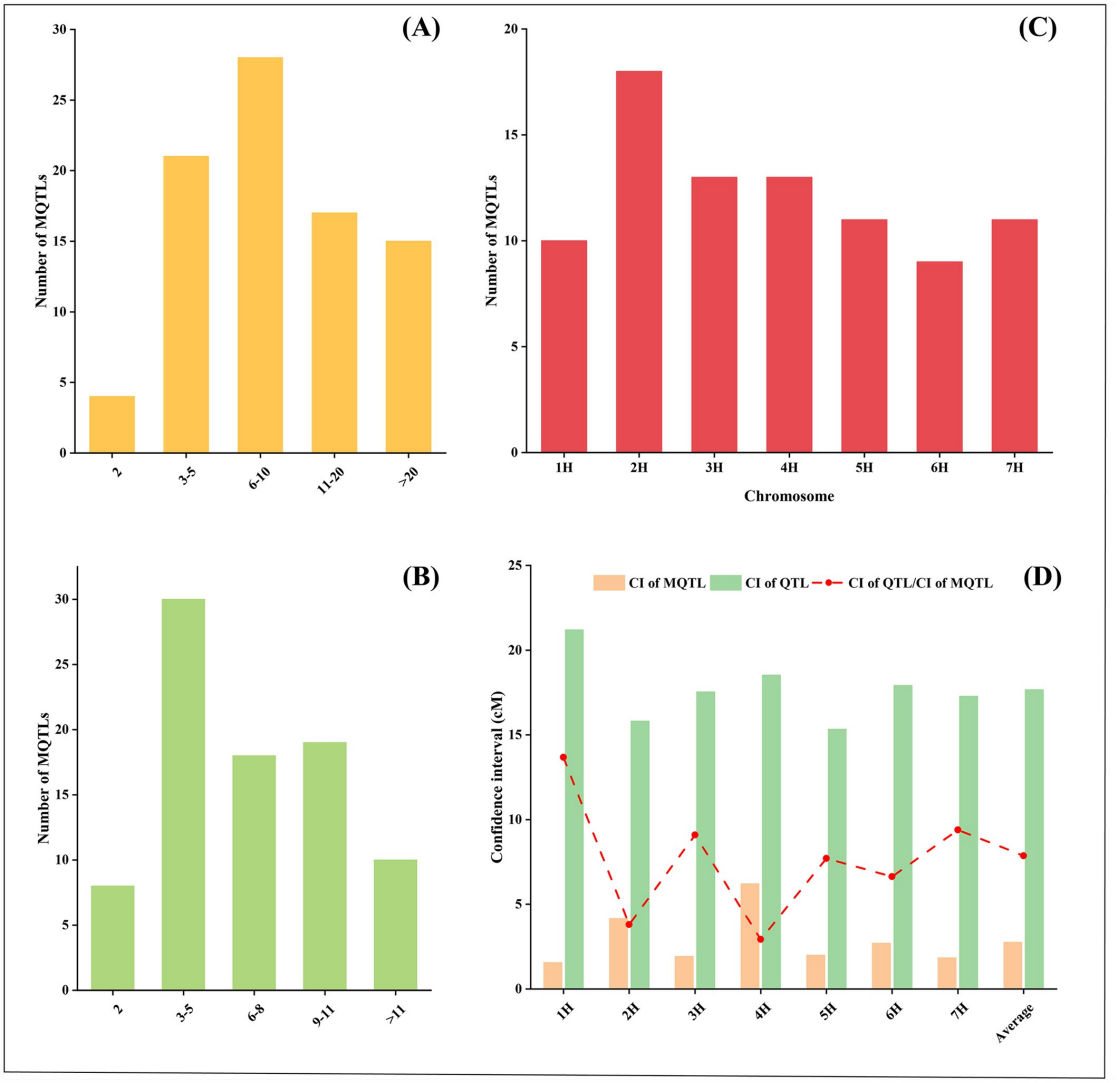
Basic information of MQTL in this study. (A) The number of MQTL containing different initial QTL numbers. (B) The number of MQTL is associated with the number of different traits. (C) MQTL distribution on seven chromosomes. (D) Confidence interval comparison between initial QTLs (green bar) and MQTLs (orange bar).

The 1080 initial QTLs identified from 58 individual studies were mapped to the consensus map, of which 1073 QTLs were integrated into 85 MQTLs by QTL meta-analysis, and the remaining 7 QTLs had no overlapping regions with the above MQTLs (S5 Table). These MQTLs contained at least 2 initial QTLs, 81 (95.29%) MQTLs contained 3 or more QTLs, and 32 (37.65%) MQTLs contained 11 or more. 6 MQTLs, MQTL2H-8 (83), MQTL2H-13 (37), MQTL3H-3 (36), MQTL4H-4 (35), MQTL5H-5 (39) and MQTL6H-5 (43), were integrated over 30 QTLs (Fig 3A). Since yield is influenced by multiple traits and numerous genes, each MQTL was associated with at least two different yield traits, 35.29% (30/85) of the MQTLs contained QTLs for 3–5 different yield-related traits and 55.29% (47/85) of the MQTLs affected 6 and more different yield traits (Fig 3B). The 85 MQTLs were unevenly distributed on seven chromosomes, and the number of MQTLs on each chromosome ranged from 9 (6H) to 18 (2H), which was generally consistent with the distribution of the initial QTL on the chromosomes (Fig 3C).

The CI of MQTL on different chromosomes varied between 1.55 cM (1H) and 6.21 cM (4H) with an average CI of 2.76 cM, which was 7.86-fold narrower than the mean CI of the initial QTL (Fig 3D). The reduction in the CI of MQTL on different chromosomes differed significantly, with the mean CI of MQTL on chromosomes 2H and 4H narrowing by 3.8 and 2.93-fold, respectively, and the mean CI of MQTL on chromosomes 1H and 7H narrowing by 13.67 and 9.39-fold, respectively (Fig 3D).

### Validating MQTL with GWAS

**Fig 4:**
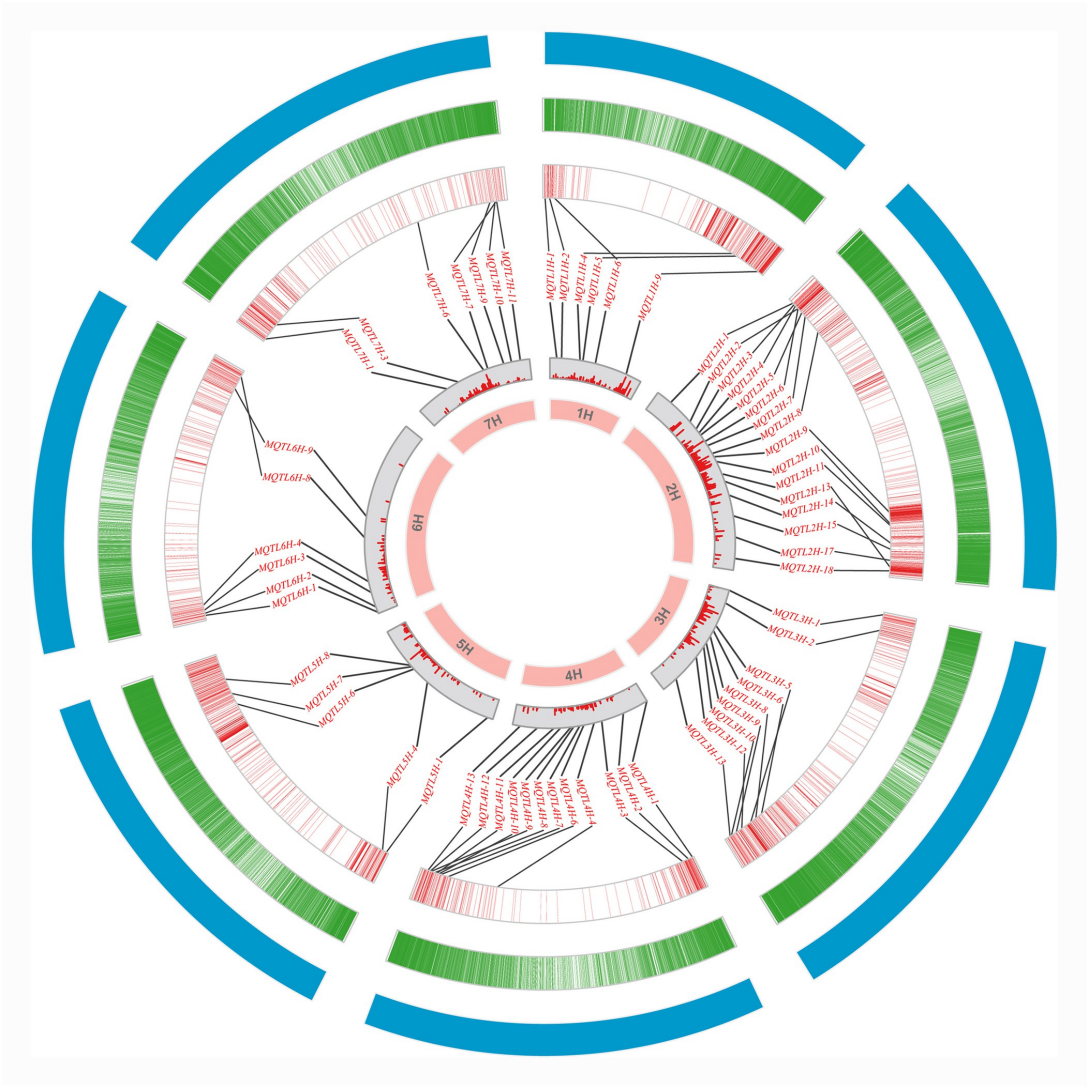
Distribution of MQTL on chromosomes verified by GWAS. The circles from inside to outside indicate the genetic map, the PVE of the initial QTL, the position of the MTA on the physical map, the high-confidence gene distribution, and the physical map, respectively.

The 85 MQTLs were mapped to the barley reference genome, of which 68 (80%) MQTLs had physical intervals less than 20 Mb (S5 Table). To verify the accuracy of these MQTLs, the physical locations of GWAS-MTAs concerning yield-related traits in barley for the last 6 years were compared with the physical locations of the above MQTLs. MQTLs with overlapping physical locations with MTAs were considered to be co-localized. Of the 68 MQTLs, 61 were validated in at least one of the 16 GWAS studies, and 29 MQTLs were detected at least four times, with MQTL1H-9, MQTL2H-3, MQTL2H-6, MQTL3H-1, and MQTL4H-12 detected no less than 8 times (S6 Table). Furthermore, these MQTLs were distributed in clusters or nested on chromosomes, such as MQTL2H-1 (9.42–18.67 Mb), MQTL2H-3 (13.21–29.98 Mb), and MQTL2H-4 (17.33–19.64 Mb) on chromosome 2H, and MQTL4H-12 (591.67–597.83 Mb) and MQTL4H-13 (597.08–606.93 Mb) on chromosome 4H (Fig 4).

**Fig 5:**
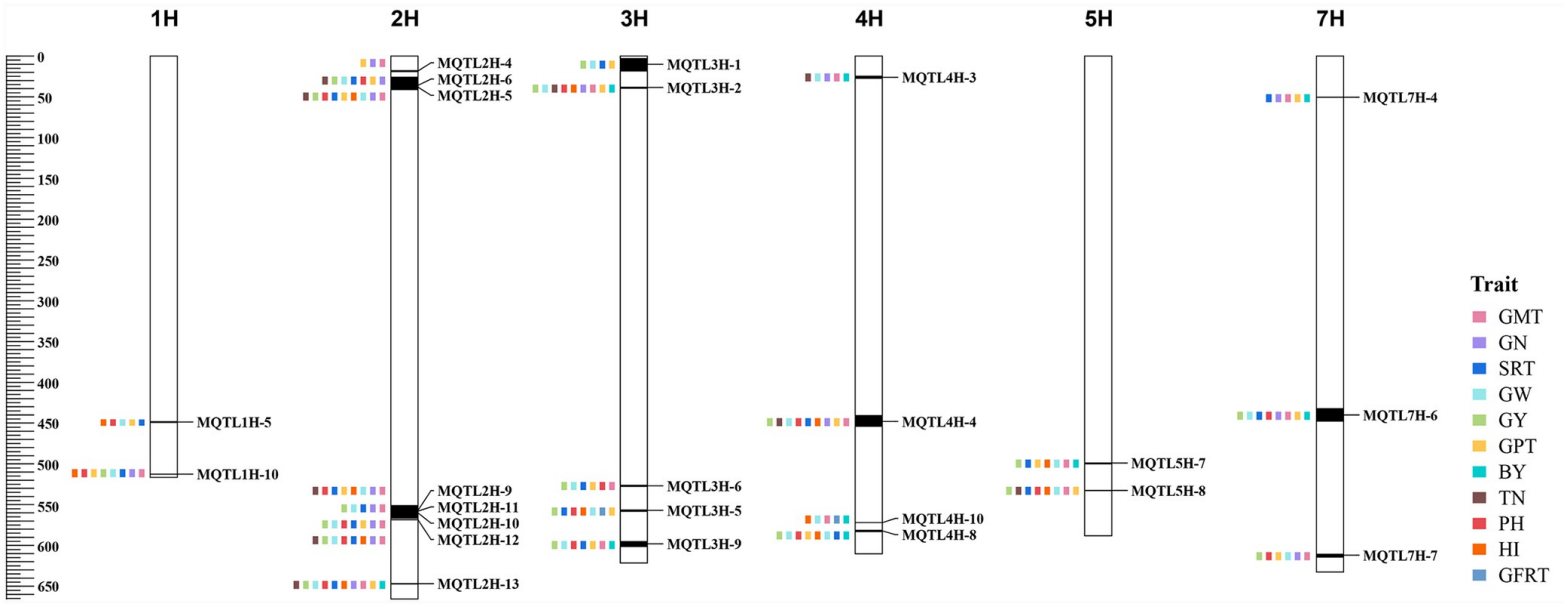
Distribution of 25 breeder’s MQTLs affecting different yield-related traits on chromosomes. The axes on the left indicate physical distances (Mb), and different traits were represented by squares of different colors. GMT grain morphological traits, GN grain number, SRT spike-related traits, GW grain weight, GY grain yield, GPT growth period traits, BY biomass yield, TN tiller number, PH plant height, HI harvest index, GFRT grain filling-related traits.

Twenty-five breeder’s MQTLs were screened among the MQTL validated in the GWAS study according to the criteria developed by Löffler et al [34]. These breeder’s MQTLs were largely distributed at the ends of chromosomes with high gene density (Fig 5; Table 1). Grain number, thousand grain weight, and spike number were considered as yield component traits, and some breeder’s MQTLs were associated with these traits simultaneously, such as MQTL1H-10, MQTL2H-5, MQTL2H-6, MQTL2H-10, MQTL2H-12, MQTL2H-13, MQTL4H-4, and MQTL7H-4. In addition, the breeder’s MQTLs associated with plant height and tiller number traits important for yield were mainly distributed on both ends of chromosome 2H, the beginning of chromosome 3H, and the ends of 4H and 5H (Fig 5; Table 1).

**Table 1:** The 25 breeder’s MQTLs for yield and yield-related traits in barley.

MQTL Name	Chr^a^	Position /cM	CI/cM^b^	Flanking marker	Physical interval/Mb	Num. of QTL	Trait^c^
MQTL1H-5	1H	54.03	53.67–54.59	1_0006–2_0990	447.79–449.73	8	GPT(2), SRT(2), PH(2), HI(1), GW(1)
MQTL1H-10	1H	135.16	135.11–135.21	GBM1461—bPb-2260	512.54–513.02	29	GMT(14), GN(2), HI(2), GPT(2), SRT(5), PH(2), GW(1), GY(1)
MQTL2H-4	2H	94.15	93.32–94.99	3_1534—bPb-8354	17.33–19.64	4	GMT(2), GPT(1), GN(1)
MQTL2H-5	2H	98.07	97.75–98.4	BOPA2_12_10847—SCRI_RS_110647	37.53–38.25	19	GMT(1), GN(1), GW(3), HI(2), GPT(3), SRT(4), PH(2), TN(1), GY(2)
MQTL2H-6	2H	110.51	110.17–110.85	MWG858–2_1015	25.38–41.62	20	GN(4), GPT(6), PH(1), SRT(3), GW(2), TN(1), GY(3)
MQTL2H-9	2H	139.54	138.85–140.24	1_1449–3_0746	555.97–558.85	19	GMT(6), GN(2), GW(4), HI(2), GPT(2), PH(1), SRT(1), TN(1)
MQTL2H-10	2H	148.04	147.44–148.51	BOPA2_12_31394–1_1214	550.59–567.09	26	GMT(6), GN(4), GPT(1), SRT(3), PH(1), GW(7), GY(4)
MQTL2H-11	2H	155.44	155.25–155.63	GBM1062—InDel2087	554.54–561.86	9	GMT(4), GN(2), SRT(1), GW(1), GY(1)
MQTL2H-12	2H	161.42	160.72–161.58	JHI-Hv50k-2016-106572—JHI-Hv50k-2016-106776	568.41–568.59	15	GMT(2), GN(5), HI(1), SRT(2), PH(1), GW(1), TN(2), GY(1)
MQTL2H-13	2H	182.43	182.25–183.31	1_1383–2_0293	646.40–647.48	37	BY(1), GPT(6), GMT(7), GN(4), HI(1), PH(4), SRT(7), GW(4), TN(1), GY(2)
MQTL3H-1	3H	0.26	0.06–0.59	2_0175–3_1448	2.55–18.85	8	GPT(1), SRT(3), GW(3), GY(1)
MQTL3H-2	3H	22.12	21.61–22.64	JHI-Hv50k-2016-164364—JHI-Hv50k-2016-164450	37.90–39.91	20	BY(1), GPT(6), GMT(1), GN(2), HI(1), PH(2), GW(1), TN(2), GY(4)
MQTL3H-5	3H	61.14	61.09–61.19	2_1341–3_1220	556.05–558.45	13	GPT(3), GFRT(1), GW(3), HI(1), PH(3), SRT(1), GY(1)
MQTL3H-6	3H	71.13	70.24–72.02	JHI-Hv50k-2016-196481—SCRI_RS_136937	525.77–527.98	10	GMT(1), GPT(4), PH(1), SRT(1), GW(2), GY(1)
MQTL3H-9	3H	93.83	92.96–94.47	2_1263—InDel3133	594.74–601.73	15	BY(2), GMT(1), GPT(6), SRT(1), PH(2), GW(2), GY(1)
MQTL4H-3	4H	80.29	80.06–80.52	SCRI_RS_6956—JHI-Hv50k-2016-232968	24.12–27.55	7	BY(1), GMT(3), GN(1), GW(1), TN(1)
MQTL4H-4	4H	92.85	92.81–92.86	1_1114–3_1435	440.17–454.32	35	GPT(9), GMT(2), GN(1), HI(2), SRT(4), PH(5), GW(6), TN(2), GY(4)
MQTL4H-8	4H	126.75	126.36–127.13	EBmac0701–1_1228	580.93–583.45	23	BY(1), SRT(5), GW(1), HI(1), GPT(2), PH(5), GW(6), GY(2)
MQTL4H-10	4H	148.34	147.69–148.98	3_0429—JHI-Hv50k-2016-262214	571.71–572.18	26	BY(1), GFRT(1), GMT(1), GW(4), HI(2), GPT(3), SRT(6), PH(5), TN(1), GY(2)
MQTL5H-7	5H	157.92	157.9–157.94	bPb-6643–1_0477	498.52–500.26	18	BY(1), GMT(2), GW(4), HI(1), GPT(3), SRT(2), GY(5)
MQTL5H-8	5H	161.11	160.95–161.26	3_0222–1_0845	532.48–532.95	28	GPT(7), GMT(2), GW(4), HI(2), PH(5), SRT(1), TN(1), GY(6)
MQTL7H-4	7H	91.3	91.03–91.56	2_0165—JHI-Hv50k-2016-461918	50.45–50.46	14	BY(1), GPT(2), GMT(3), GN(1), SRT(2), GW(1), PH(1), GY(3)
MQTL7H-6	7H	123.26	123.26–123.27	2_1300—InDel7063	431.87–448.12	29	BY(1), GPT(2), GMT(12), GN(3), PH(1), SRT(3), GW(6), GY(1)
MQTL7H-7	7H	130.32	130.27–130.37	SCRI_RS_162972—JHI-Hv50k-2016-511983	610.18–614.80	17	GMT(7), GN(1), GW(3), GPT(2), PH(2), GY(2)

^a^ Chromosomes

^b^ The confidence interval (95%) of MQTL

^c^ GMT grain morphological traits, GN grain number, SRT spike-related traits, GW grain weight, GY grain yield, GPT growth period traits, BY biomass yield, TN tiller number, PH plant height, HI harvest index, GFRT grain filling-related traits.

### Candidate gene mining and expression pattern analysis based on orthologs

**Fig 6:**
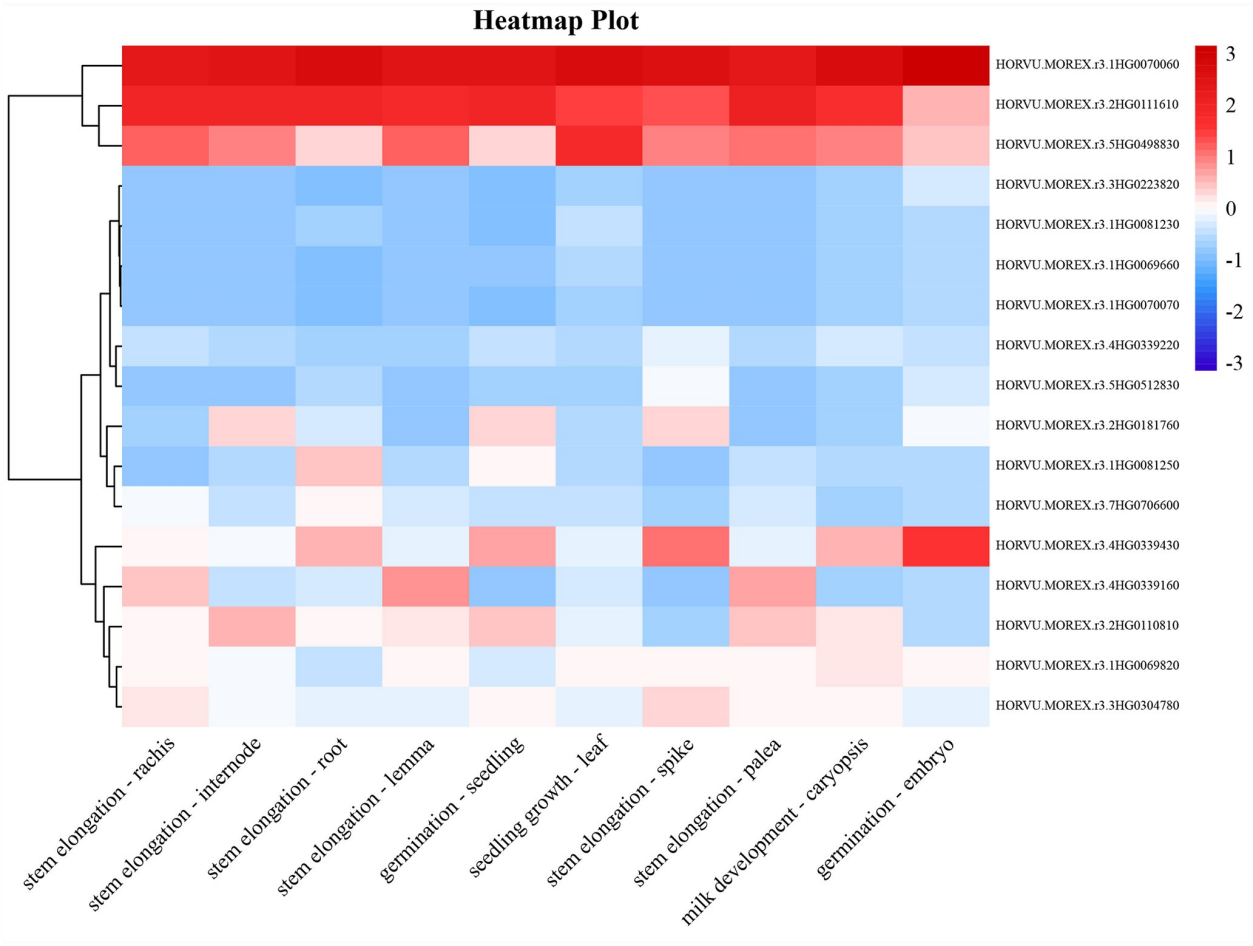
Expression characteristics of 17 candidate genes in 10 barley tissues. The expression level gradually increases from blue to red.

Nineteen high-confidence MQTLs (hcMQTL) screened out of 85 MQTLs were mined for candidate genes (CGs) according to the criteria defined by Venske et al [35]. A total of 479 gene models were identified within the region of hcMQTLs, with MQTL7H-1 detecting the largest number of 60 gene models, MQTL1H-7 and MQTL2H-12 identifying only 6 and 2 gene models, respectively, and MQTL7H-4 detecting no gene models (S7 Table). To further identify candidate genes for yield and yield-related traits in barley, a comparative genomics strategy was used to mine barley orthologs for genes related to yield-related traits in rice, wheat, and maize within the hcMQTL region. A total of 17 barley orthologs were identified in 19 hcMQTL regions, 10 of which were from maize genes, 8 from rice genes (one of which is identical to maize), and none from wheat genes (Table 2). The expression characteristics of the 17 candidate genes were analyzed in root, internode, leaf, and spike tissues of barley at different growth periods, and their expression patterns were classified into two categories (S8 Table, Fig 6). The genes in the first category were expressed at high levels in all tissues at different growth periods, including *HORVU*.*MOREX*.*r3*.*1HG0070060* (MQTL1H-5), *HORVU*.*MOREX*.*r3*.*2HG0111610* (MQTL2H-5) and *HORVU*.*MOREX*.*r3*.*5HG0498830* (MQTL5H-7). Most of the genes in the second category were expressed at low levels in different periods and *HORVU*.*MOREX*.*r3*.*4HG0339430 (MQTL4H-3)* was expressed at high levels in the embryo and spike tissues at the germination and stem elongation stages.

**Table 2:** Barley orthologs of yield-associated genes in rice and maize within the hcMQTLs region.

HcMQTL^a^	Barley orthologs	Position (Mb)	Species	Gene ID	Gene name	Regulated Traits	Description	Reference
*MQTL1H-5*	*HORVU*.*MOREX*.*r3*.*1HG0069820*	448.81	Maize	*Zm00001d012544*	*myb74*	flowering time	myb domain protein 81	[36]
*MQTL1H-5*	*HORVU*.*MOREX*.*r3*.*1HG0070060*	449.5	Maize	*Zm00001d043113* *Zm00001d008295*	*arf3* *arf4*	inflorescence development, spike length and kernel number	ADP-ribosylation factor A1E ADP-ribosylation factor A1B	[37]
*MQTL1H-5*	*HORVU*.*MOREX*.*r3*.*1HG0069660*	448.21	Rice	*LOC_Os01g59410*	*OsVQ4*	thousand grain weight	VQ motif family protein, expressed	[38]
*MQTL1H-5*	*HORVU*.*MOREX*.*r3*.*1HG0070070*	449.51	Rice	*LOC_Os03g48740*	*OsIAGLU*	tiller formation, plant height, panicle length, root length	indole-3-acetate beta-glucosyltransferase, putative, expressed	[39]
*MQTL1H-7*	*HORVU*.*MOREX*.*r3*.*1HG0081230*	488.11	Rice	*LOC_Os01g47900*	*OsLSK1*	panicle architecture and grain yield	S-locus-like receptor protein kinase, putative, expressed	[40]
*MQTL1H-7*	*HORVU*.*MOREX*.*r3*.*1HG0081250*	488.14	Rice	*LOC_Os01g47900*	*OsLSK1*	panicle architecture and grain yield	S-locus-like receptor protein kinase, putative, expressed	[40]
*MQTL2H-5*	*HORVU*.*MOREX*.*r3*.*2HG0111610*	37.74	Maize	*Zm00001d034553*	*cesa5*	kernel weight	Cellulose synthase-5	[41]
*MQTL2H-6*	*HORVU*.*MOREX*.*r3*.*2HG0110810*	33.64	Maize	*Zm00001d007195*	*abf4*	ear development	Alpha-L-arabinofuranosidase 1	[42]
*MQTL2H-11*	*HORVU*.*MOREX*.*r3*.*2HG0181760*	559.02	Rice	*LOC_Os10g33780*	*TAW1*	inflorescence development	DUF640 domain containing protein, putative, expressed	[43]
*MQTL3H-1*	*HORVU*.*MOREX*.*r3*.*3HG0223820*	10.65	Maize	*Zm00001d025355*	*incw3*	ear weight, ear grain weight, ear length and kernel length	Invertase cell wall3	[44]
*MQTL3H-5*	*HORVU*.*MOREX*.*r3*.*3HG0304780*	556.95	Maize	*Zm00001d028265*	*crr8*	root morphology	Response regulator 8%253B Two-component response regulator ARR1	[45]
*MQTL4H-3*	*HORVU*.*MOREX*.*r3*.*4HG0339220*	25.45	Maize	*Zm00001d033869*	*emp4*	seedling development	empty pericarp4	[46]
*MQTL4H-3*	*HORVU*.*MOREX*.*r3*.*4HG0339430*	26.19	Maize	*Zm00001d033876*	*grftf1*	Inflorescence development	Growth-regulating factor 2	[47]
*MQTL4H-3*	*HORVU*.*MOREX*.*r3*.*4HG0339160*	25	Rice	*LOC_Os03g51740*	*OsACS1*	internode elongation	aminotransferase, classes I and II, domain containing protein, expressed	[48]
*MQTL4H-3*	*HORVU*.*MOREX*.*r3*.*4HG0339430*	26.19	Rice	*LOC_Os03g51970*	*OsGRF6*	spikelets development	growth-regulating factor, putative, expressed	[49]
*MQTL5H-7*	*HORVU*.*MOREX*.*r3*.*5HG0498830*	499.52	Maize	*Zm00001d021291*	*cct41*	flowering time	Two-component response regulator-like APRR9	[50]
*MQTL5H-8*	*HORVU*.*MOREX*.*r3*.*5HG0512830*	532.66	Maize	*Zm00001d034163*	*dof25*	leaf development	Dof zinc finger protein DOF5.7	[51]
*MQTL7H-6*	*HORVU*.*MOREX*.*r3*.*7HG0706600*	439.16	Rice	*LOC_Os08g01670*	*OsPMEI28*	plant height and culm diameter	invertase/pectin methylesterase inhibitor family protein, putative, expressed	[52]

## Discussion

With the increasing world population and variable climatic conditions, improving yield remains the primary goal of barley breeding. Over the last two decades, a large number of genetic studies have been carried out to identify QTL for yield and yield-related traits in barley (S1 Table). However, using these data for breeding programs remains difficult. In this regard, QTL meta-analysis is available to identify MQTL affecting different yield traits in barley. A previous study on meta-QTL analysis of yield-related traits in barley collected 639 initial QTLs for grain yield, thousand grain weight, plant height, heading date, and number of ears per m^2^ traits from 26 mapping populations [24]. Nevertheless, the accuracy of the QTL meta-analysis was significantly and positively correlated with the number of initial QTLs [53]. Many barley yield-related QTLs have been sequentially identified and published in recent years [10, 54–57]. Therefore, it is particularly important to integrate the newest QTL data to identify more stable MQTLs. In this study, a total of 1080 initial QTLs for 11 yield-related traits such as grain yield, grain morphology, grain number, spike trait, grain weight, growth period traits, plant height, etc. were collected from 80 different mapping populations for QTL meta-analysis. In comparison, this work used more initial QTLs and covered more yield-related traits, making it the most comprehensive study of QTL meta-analysis for barley yield-related traits to date.

QTL meta-analysis eliminates the effects of environment, genotypes, and molecular marker types, and integrates QTL from different genetic backgrounds to obtain more stable and reliable MQTL [13]. For the current study, we used 1080 initial QTLs information to identify MQTLs reported in about 58% of previous MQTL studies for yield-related traits in barley [24]. Among these yield-related traits, QTL for thousand grain weight (TGW), plant height (PH), heading date (HD), and grain yield (GY) were the most frequently QTLs observed in MQTL. More than 60% of these MQTLs were associated with TGW, PH and HD (S5 Table). QTL for HD, TGW, and YD were also the most frequent among MQTL in the previous barley MQTL study [24]. The high frequency of QTLs for these agronomic traits is probably due to the ease of measurement of the traits, which are often reported in different QTL studies. Moreover, these traits are generally regulated by multiple genes and may carry different combinations of alleles in genetic populations from different backgrounds [58–60]. In this study, 32 (37.65%) MQTLs consisted of at least 11 initial QTLs, of which 6 consisted of more than 30 QTLs (Fig 3A), and the frequency of MQTLs with a high number of initial QTLs was significantly higher than in previous studies [24]. Comparatively, this study greatly improved the accuracy of identifying genetic regions controlling yield-related traits in barley.

These MQTLs were unevenly distributed on seven chromosomes, with the highest number of MQTLs on chromosome 2H (18) and the lowest on chromosome 6H (9), which was generally consistent with the distribution of the initial QTL on the chromosomes (Fig 3C). The distribution was inconsistent with the previously reported MQTLs for yield-related traits in barley, which were similar in form to the initial QTL [24]. In addition, the initial QTL and MQTL in this study were mainly distributed in the sub-telomeric region of chromosomes (Fig 4; S5 Table). The distribution density of QTL is mainly relates to gene density, functional locus polymorphism rate, and recombination rate [61]. The number of genes and recombination rate in the chromosome sub-telomeric region were the highest in the barley genome [62, 63], which was consistent with the results of the distribution density of QTL in this study, with similar results in wheat and rice studies [17, 64, 65]. Comparing the physical locations of the MQTLs in this study with those found in previous QTL meta-analyses [24], 15 of the 31 MQTLs were found to overlap with the MQTL locations identified in this study, further confirming that the QTL meta-analysis in this study was accurate and reliable. The QTL meta-analysis integrating the initial QTL significantly narrowed the confidence intervals of the QTL. The average CI of MQTLs on different chromosomes was 2.76 cM, which was 7.86-fold narrower than the initial QTL (Fig 3D). Correspondingly, the physical intervals of MQTLs were further reduced, providing a basis for aggregation of important QTLs in barley breeding and candidate gene prediction.

In addition to traditional QTL linkage analysis, genome-wide association studies based on linkage disequilibrium have been widely used to identify QTL for quantitative traits in various crops [25–27]. Currently, verification of MQTL results using GWAS information has been reported in QTL meta-analysis in rice and wheat [17, 19, 20]. Khahani et al. [24] validated 17 MQTLs associated with yield traits in barley using GWAS results. In this study, 61 MQTLs were validated in at least one GWAS study, and 29 MQTLs were co-localized with MTAs in no less than four studies on GWAS results for yield-related traits published over the past five years in barley, suggesting that several candidate genes regulating barley yield might exist in these genomic regions. These MQTLs were distributed in clusters or nested on the chromosomes and mostly near the sub-telomeric region (Fig 4), which was generally consistent with previous MQTL studies for yield-related traits in wheat and barley [24, 66]. Furthermore, 25 breeder’s MQTLs were screened among the GWAS-validated MQTLs, among which eight breeder’s MQTLs were identified on chromosomes 1H, 2H, 4H, and 7H, which simultaneously affect the yield component traits of spike number, grains per spike, and thousand grain weight (MQTL1H-10, MQTL2H-5, MQTL2H-6, MQTL2H-10, MQTL2H-12, MQTL2H-13, MQTL4H-4, MQTL7H-4). Also, some breeder’s MQTLs were also identified for traits affecting plant height and tiller number (Fig 5; Table 1). Ten of the breeder’s MQTLs contained at least 20 initial QTLs from different studies, for example, MQTL2H-13 involved 37 QTLs and MQTL4H-4 involved 35 QTLs. These breeder’s MQTLs could be used for molecular marker-assisted selection to improve yield in barley.

Several important genes known to be associated with yield-related traits in barley have been identified in MQTL regions. These genes mainly regulate three different types of traits, the first of which affects flowering time in barley, including the genes *ELF3* in MQTL1H-10 [67], *Ppd-H1* in MQTL2H-6 [68], *LUX* in MQTL3H-10 [69], *PhyA* in MQTL4H-3 [70], *HvPhyC* and *Vrn-H1* in MQTL5H-8 [71, 72], *Vrn-H3* in MQTL7H-3 [73]; the second class controls row type and spikelet development in barley, including the genes *Vrs1* in MQTL2H-10 [74], *Vrs4* in MQTL3H-2 [75], *Vrs5* in MQTL4H-2 [76], and *Vrs2* in MQTL5H-7 [77]; the third class is the semi-dwarf gene *sdw1/denso* located in the MQTL3H-5 region, which controls barley plant height [78]. In addition, previous studies have reported that the genes *Ppd-H1*, *Vrs1*, *Vrs5*, and *sdw1/denso* affect leaf size, grain size, thousand grain weight, and tiller number [79–82]. These results indicated that the above genes might have pleiotropic or tightly linked effects on yield-related traits such as grain morphology, growth period, plant height, and tiller number in barley.

As barley and rice, wheat, and maize are Gramineae crops, orthologs can retain the same functions as the original genes, and analyzing their orthologs is an effective strategy for discovering important genes in barley. For example, the rice gene *GW2*, which encodes a RING-type E3 ubiquitin ligase, regulates grain width and weight [83], and the barley ortholog *HvYrg1* also affects 1000-grain weight and grain morphology [84]. Additionally, some genes related to yield-related traits in rice, maize, and wheat have similar functions in barley, such as *Ppd-H1* [68, 85, 86], *HvRA2* [75, 87], and *HvTB1* [76]. Therefore, the identification of important genes in barley based on orthology analysis is feasible.

In this study, a total of 17 orthologs related to yield traits in rice and maize were identified within 19 hcMQTL regions, 10 of which were from maize genes and 8 from rice genes (one of which was identical to maize) (Table 2), which could be potential candidate genes affecting yield and yield-related traits in barley. The barley ortholog of the rice gene *OsIAAGLU*, *HORVU*.*MOREX*.*r3*.*1HG0070070*, located in the MQTL1H-5 region, encodes glycosyltransferase. The *OsIAAGLU* gene encoding an IAA-conjugating enzyme increased the number of tillers and decreased plant height in rice under overexpression or exogenous IAA treatment [39]. Therefore, the *HORVU*.*MOREX*.*r3*.*1HG0070070* gene may be a candidate gene affecting yield-related traits in barley. The barley orthologs of the rice *OsLSK1* gene, *HORVU*.*MOREX*.*r3*.*1HG0081230* and *HORVU*.*MOREX*.*r3*.*1HG0081250*, are located in the MQTL1H-7 region and encode serine/threonine-protein kinases. Overexpression of *OsLSK1* genes encoding S-domain receptor-like kinase improved panicle architecture and grain yield in rice [40]. Thus, *HORVU*.*MOREX*.*r3*.*1HG0081230* and *HORVU*.*MOREX*.*r3*.*1HG0081250* are reliable candidate genes involved in the regulation of barley yield. The maize gene *grftf1* and the rice gene *OsGRF6* corresponding to the barley ortholog are both located in the MQTL4H-3 region of *HORVU*.*MOREX*.*r3*.*4HG0339430*, which encodes a growth-regulating factor. The gene grftf1 encoding growth-regulating factor 2 controls ear inflorescence architecture and floral development by regulating maize hormone biosynthesis [47]. The gene *OsGRF6* encodes a growth-regulating factor, which promotes growth hormone biosynthesis and regulates auxin branch and spikelet development, thereby increasing rice yield [49]. Therefore, we hypothesized that *HORVU*.*MOREX*.*r3*.*4HG0339430* is an important candidate gene for improving barley yield. Meanwhile, several barley orthologs of rice and maize genes controlling traits such as flowering time, thousand grain weight, ear length, and plant height were detected in the hcMQTL region (Table 2). These barley orthologs of yield and yield-related genes identified from rice and maize provide new information on the genetic mechanism of yield-related traits in barley.

## Materials and methods

### Collection of QTL data

An exhaustive collection and screening of the published QTL for yield and compositional traits in barley since 2001 identified a total of 54 papers that could be used for QTL meta-analysis. The basic information of these studies including the parents of the population, population type and size, traits, and molecular marker types were listed in the S1 Table.

The initial QTL collected were mainly related to yield and yield-related traits, including 11 trait categories: grain morphological traits (GMT) (grain area, grain diameter, grain length, grain perimeter, grain thickness, grain volume and grain width), grain number (GN) (grain density, grain number per plant, grain number per spike and grain number per square meter), spike-related traits (SRT) (fertile spike number, number of spike, number of spikelets, number of spikes per plant, number of spikes per square meter, spike density, spike length, spikelet number per spike and spikes per line), grain weight (GW) (grain weight per square meter, grain weight per plant, grain weight per spike and thousand grain weight), grain yield (GY) (grain yield per plant and yield), grain filling-related traits (GFRT) (grain filling period and grain filling rate), growth period traits (GPT) (days to maturity, flowering date and heading date), biomass yield (BY), plant height (PH), tiller number (TN) and harvest index (HI).

Information for the initial QTL including associated traits, flanking markers, LOD values, and phenotypic variation explained (PVE) or R^2^ value was listed in the S2 Table, with 3 and 10% assumed for the few QTLs with missing LOD and R^2^ values [35]. In addition, the confidence intervals (CI) of the initial QTL needed to be recalculated using the following equations: (1) CI = 287 / (n × PVE); (2) CI = 163 / (n × PVE); (3) CI = 530 / (n × PVE), n is the population size, and the equations were applied to the double-haploid (DH) population, recombinant inbred line (RIL) population, and F_2_ and backcross (BC) populations, respectively [88, 89].

### Construction of consensus linkage map

Six high-quality genetic linkage maps containing different molecular marker types were used to develop consensus maps: (1) barley consensus map with 2935 marker loci (2085 DArT, 850 SSR, RFLP and STS loci) [90]; (2) Barley consensus map with 775 SSR loci [91]; (3) an integrated map for barley with 2943 SNP marker loci [92]; (4) barley consensus map with 6976 molecular markers [93]; (5) linkage map of 14626 loci based on the "50K Illumina Infinium iSelect Genotyping Array" [94]; (6) consensus map with 1704 marker loci [24]. Map information from other individual studies was also used to construct the common map. The above six linkage maps were integrated using the R package LPmerge to develop consensus maps [33]. LPmerge uses linear programming to efficiently minimize the average absolute error between consensus maps and linkage maps from each group. In case of inconsistency in the order of tokens between the chain graphs, the minimum order constraint set was removed to resolve the conflict.

### QTL meta-analysis

Upon construction of the consensus linkage map in barley, the initial QTL were mapped to the consensus map utilizing BioMercator V4.2 software, followed by QTL meta-analysis on each chromosome using the Veyrieras two-step algorithm [95]. In the first step, the most prevalent value was calculated based on five models: Akaike information criterion (AIC), AIC correction (AICc), AIC 3 candidate models (AIC3), Bayesian information criterion (BIC) and average weight of evidence (AWE) considered as the optimal number of MQTL on each chromosome. In the second step, the genetic position and confidence interval of the MQTL for each chromosome were determined based on the optimal number of MQTL. The LOD score and PVE of the MQTLs were obtained by calculating the average of the LOD scores and PVE values of the initial QTL. The MQTL criteria proposed by Löffler et al. [34] for breeding programs (called breeder’s MQTL): CI < 2 cM, PVE > 10% from no less than 4 initial QTLs from different studies were followed.

The flanking marker nucleotide sequence of the MQTL was BLASTed against the barley reference genome sequence (MorexV3) to obtain the physical position of the MQTL [63]. Sequences of SSR, RFLP, STS, and AFLP markers were retrieved from grain genes (https://wheat.pw.usda.gov/GG3), DArT marker sequences were searched from the website https://www.diversityarrays.com, and SNP marker sequences were available from Close et al. [92] and Barleymap [96].

### Evaluating the validity of MQTL with GWAS

Information on significant marker-trait associations (MTAs) identified in 16 GWAS studies published in the last five years for yield and related traits in barley was collected to assess the validity of the MQTLs in this study. Relevant details from these GWAS papers, such as population size, traits, type and number of markers, and number of MTAs, were listed in the S9 Table. The physical locations of MTAs were obtained from the source files or Barleymap [96], and MQTL that overlapped with at least one MTA was considered GWAS-validated MQTL.

### Candidate gene mining and expression pattern analysis within the hcMQTL region

High confidence MQTL (hcMQTL) with genetic distance less than 1 cM, physical distance less than 20 Mb, and containing at least five initial QTL were considered for candidate gene (CGs) mining according to the criteria defined by Venske et al. [35]. The physical interval of hcMQTL less than 2 Mb was directly identified for CGs, while the rest of the peak hcMQTL was calculated according to the formula developed by Saini et al. [66], and then CGs were detected within the peak ± 1 Mb region. The information of candidate genes in each hcMQTL was searched using the Locate by position tool in the Barleymap database [96]. Considering rice, wheat, maize, and barley as the same gramineous crops, a comparative genomics strategy was utilized to mine orthologs of rice, wheat, and maize within the hcMQTL region of the barley genome using the Ensembl plant database (http://plants.ensembl.org/). The China Rice Data Center (www.ricedata.cn/), Maize Genetics and Genomics Database (https://www.maizegdb.org/), and WheatOmics 1.0 [97] were searched for information on yield-related genes in rice, maize, and wheat, respectively, to find their orthologs in the hcMQTL region and identify candidate genes for yield-related traits in barley. The expression levels of the above candidate genes in various tissues of barley were further analyzed with Genevestigator software, including expression data of seedling, root, internode, leaf, spike, and caryopsis tissues during germination, stem elongation, and milk development. In addition, heat maps of the expression patterns of candidate genes in different tissues were plotted using Origin 2021 software.

## Conclusions

In summary, we integrated previous QTL for barley yield and yield-related traits by constructing a high-density consensus map, aiming to identify yield-related MQTL, hcMQTL, and candidate genes to better provide useful information for barley high-yield breeding. A total of 85 MQTLs were identified in this study, of which 61 MQTLs were validated in GWAS studies. Among these 61 MQTLs, 25 breeder’s MQTLs were screened and could be used for molecular marker-assisted selection to improve barley yield. In addition, 17 barley orthologs of rice and maize yield-related genes were detected in the hcMQTL region based on a comparative genomics strategy, and these genes may be potential candidates for influencing barley yield. The results of this study will further enhance our understanding of the genetic mechanism of barley yield and lay the foundation for mining reliable candidate genes.

## Supporting information

S1 Table Basic information on QTL studies for yield and yield-related traits in barley used for QTL meta-analysis. (XLSX)

S2 Table Initial QTL information for QTL meta-analysis. (XLSX)

S3 Table Construction of consensus map for barley in this study. (XLSX)

S4 Table Genetic distance and number of markers for consensus map. (XLSX)

S5 Table Details of MQTL for yield and yield-related traits in barley. (XLSX)

S6 Table Comparison of MQTLs with important marker-trait associations (MTAs) in previous GWAS for yield and related traits in barley. (XLSX)

S7 Table Gene annotation information in hcMQTL region. (XLSX)

S8 Table Expression of 17 candidate genes screened within the hcMQTL region across 10 barley tissues. (XLSX)

S9 Table Information of GWAS for yield-related traits in barley used to validate MQTL. (XLSX)

S1 File (XLSX)
